# Supplementary material for: Exploring the preferences of multiple stakeholder groups for family involvement in early intervention services for psychosis: A cross-sectional mixed-methods study
Source: PLOS Ment Health. 2026 Apr 10;3(4):e0000430. doi: 10.1371/journal.pmen.0000430 (PMC13068328; doi:10.1371/journal.pmen.0000430)
Supplement: S2 Table — (DOCX) [file pmen.0000430.s002.docx]

**Supplementary Table 2: Statements under each question and individual ranks of all participants**

| **2a. In what ways should families/ carers of persons with psychosis be involved?** | **PT1** | **PT2** | **PT3** | **FM1** | **FM2** | **FM3** | **CL1** | **CL2** | **CL3** |
| --- | --- | --- | --- | --- | --- | --- | --- | --- | --- |
| Families can have the young person with psychosis live with them. | 5 | 2 | 10 | 1 | 1 | 10 | 9 | 4 | 9 |
| Families can support during crises, relapses, or hospitalizations. | 1 | 5 | 2 | 2 | 2 | 3 | 1 | 3 | 4 |
| Families can accompany the young person during appointments at the clinic. | 9 | 10 | 5 | 4 | 10 | 6 | 7 | 10 | 6 |
| Families can support the young person with their work or school. | 6 | 9 | 9 | 10 | 9 | 9 | 10 | 5 | 10 |
| Families can update the treating team about progress and concerns such as changes in young family member’s behavior, so that treatment can be adjusted. | 4 | 4 | 6 | 5 | 4 | 4 | 3 | 8 | 5 |
| Families can educate themselves about the illness. | 3 | 1 | 7 | 3 | 5 | 2 | 8 | 7 | 2 |
| Families can develop emotional acceptance and adjust their expectations from their young family member. | 7 | 8 | 8 | 7 | 8 | 5 | 4 | 2 | 1 |
| Families can help the young person to stay in treatment and be in contact with the treating team. | 8 | 7 | 4 | 8 | 6 | 1 | 6 | 6 | 3 |
| Families can be aware of and be involved in developing treatment plans. | 10 | 3 | 3 | 9 | 3 | 8 | 2 | 9 | 7 |
| Families can offer emotional support. | 2 | 6 | 1 | 6 | 7 | 7 | 5 | 1 | 8 |
|  | | | | | | | | | |
| **2b. What influences the involvement of families/carers of persons with psychosis?** | **PT1** | **PT2** | **PT3** | **FM1** | **FM2** | **FM3** | **CL1** | **CL2** | **CL3** |
| Involvement is influenced by each family’s culture around autonomy and support. | 7 | 8 | 9 | 1 | 8 | 2 | 7 | 9 | 4 |
| Involvement is influenced by the resources of families such as finances, accessibility of the clinic, time available based on job and other commitments, etc. | 5 | 2 | 4 | 3 | 10 | 8 | 8 | 8 | 3 |
| Involvement is influenced by the age or development of patients (e.g., adolescents versus young adults versus adults). | 6 | 9 | 10 | 2 | 9 | 6 | 6 | 4 | 2 |
| There should always be some involvement of families/carers in treatment. | 8 | 5 | 7 | 4 | 1 | 3 | 10 | 10 | 9 |
| There should always be some involvement of families/carers in treatment, except when the families/carers are unhelpful or harmful. | 9 | 6 | 1 | 6 | 2 | 1 | 3 | 2 | 6 |
| The young person’s consent is necessary for families/carers to be involved in treatment. | 3 | 3 | 2 | 7 | 6 | 5 | 1 | 1 | 8 |
| The need for and frequency of family contact depends on the phase of recovery, e.g., more contact when there is a crisis or relapse and less contact when the person is doing well. | 4 | 1 | 6 | 5 | 4 | 9 | 5 | 7 | 5 |
| The frequency and types of involvement of families should be discussed jointly by patients, families and treating teams. | 1 | 4 | 5 | 8 | 3 | 4 | 2 | 5 | 1 |
| The frequency and types of involvement of families should be set based on patients’ preferences. | 2 | 7 | 3 | 9 | 7 | 7 | 4 | 3 | 7 |
| When patients are doing well, it is okay for busy treating teams to not contact families. | 10 | 10 | 8 | 10 | 5 | 10 | 9 | 6 | 10 |
|  | | | | | | | | | |
| **2c. How often should families/carers be involved?** | **PT1** | **PT2** | **PT3** | **FM1** | **FM2** | **FM3** | **CL1** | **CL2** | **CL3** |
| Families should be present at every point in treatment. | 1 | 9 | 9 | 1 | 8 | 4 | 3 | 10 | 7 |
| Families and the treating team should have contact with each other at least once a week for the first month. | 6 | 3 | 3 | 7 | 3 | 1 | 5 | 9 | 6 |
| Families and the treating team should have contact with each other at least once a month throughout treatment (which is usually for 2 years at *). | 7 | 1 | 5 | 3 | 7 | 2 | 2 | 8 | 5 |
| Over the course of two years, there should be a minimum number of times that treatment teams should contact families. Beyond this minimum, treatment teams can also increase contact depending on patients’ needs. | 8 | 2 | 7 | 2 | 4 | 8 | 4 | 2 | 2 |
| Over the course of two years, there should not be a minimum number of times that treatment teams should contact families. Instead, they should contact families as and when needed. | 3 | 7 | 4 | 9 | 6 | 9 | 7 | 5 | 10 |
| Families and treating teams should be jointly responsible for maintaining contact with each other. | 5 | 5 | 2 | 4 | 2 | 3 | 6 | 4 | 3 |
| Maintaining contact with the family should be the primary responsibility of the treatment team (with families having the option of initiating contact). | 4 | 8 | 6 | 6 | 5 | 5 | 8 | 3 | 8 |
| Programs like * should systematically record presence or absence of contact with families in each patient’s chart. | 9 | 6 | 8 | 5 | 9 | 6 | 10 | 1 | 4 |
| There should be guidelines about involving families for treatment teams to follow. | 10 | 4 | 10 | 10 | 1 | 7 | 1 | 7 | 1 |
| Because each person’s situation is different, there cannot be any common guidelines about involving families in treatment. | 2 | 10 | 1 | 8 | 10 | 10 | 9 | 6 | 9 |
|  | | | | | | | | | |
| **2d. How should consent and confidentiality be dealt with in involving families/carers?** | **PT1** | **PT2** | **PT3** | **FM1** | **FM2** | **FM3** | **CL1** | **CL2** | **CL3** |
| If a patient is not a threat to himself or others, there should be no insistence on involving families/carers if the patient does not desire such involvement. | 1 | 7 | 1 | 2 | 8 | 7 | 9 | 6 | 8 |
| If the patient does not consent to their treatment provider sharing information with families/carers, the treating team can still receive information or updates from families and can share general information about the illness and treatment if families contact them. | 6 | 6 | 3 | 3 | 4 | 3 | 10 | 5 | 1 |
| If the patient does not consent to their treatment provider sharing information with families/carers, the treating team should not have any contact with families/carers. | 7 | 10 | 10 | 8 | 7 | 10 | 2 | 9 | 10 |
| Even if the patient has consented for families to be involved, treatment providers should always check with patients before disclosing any specific information. | 3 | 5 | 4 | 4 | 10 | 4 | 8 | 7 | 5 |
| When the **patient has consented f**or families to be involved, treatment providers should use their judgment in deciding what information should and should not be disclosed to families. | 5 | 1 | 8 | 5 | 1 | 9 | 5 | 2 | 3 |
| Laws and regulations around consent and confidentiality make it difficult to involve families. | 8 | 9 | 9 | 1 | 9 | 6 | 7 | 10 | 9 |
| When a patient **does not consent to** involving families/carers, treating teams should try to convince them that family support can be helpful and discuss their concerns about family involvement. | 4 | 2 | 7 | 7 | 3 | 5 | 1 | 3 | 7 |
| It is possible to involve families/carers and also respect patient consent and confidentiality. | 2 | 4 | 2 | 9 | 5 | 2 | 4 | 4 | 2 |
| Even when patients consent for families to be involved, patients themselves should make key treatment decisions. | 9 | 8 | 5 | 10 | 6 | 8 | 3 | 1 | 4 |
| When patients consent for families to be involved, key treatment decisions should involve both the patient and the family. | 10 | 3 | 6 | 6 | 2 | 1 | 6 | 8 | 6 |
| **Legend:**  PT-Patient, FM-Family Member, CL-Clinician  *Name of the early psychosis intervention program redacted for confidentiality purposes | | | | | | | | | |
