# Supplementary material for: Exploring the preferences of multiple stakeholder groups for family involvement in early intervention services for psychosis: A cross-sectional mixed-methods study
Source: PLOS Ment Health. 2026 Apr 10;3(4):e0000430. doi: 10.1371/journal.pmen.0000430 (PMC13068328; doi:10.1371/journal.pmen.0000430)
Supplement: S3 Table — (DOCX) [file pmen.0000430.s003.docx]

**Supplementary Table 3: Statements on which consensus was not reached that did not qualify under any of the importance categories based on themes (n=18)**

| **3a. Question 1: Types of family involvement** | **Stakeholder Endorsement (n= 9)** |
| --- | --- |
| Families can have the young person with psychosis live with them. | 1PT+2FM= Most important  1PT+1CL = Moderately important  1PT+1FM+2CL= Least important |
| Families can develop emotional acceptance and adjust their expectations from their young family member. | 2CL = Most important  1PT+2FM+1CL= Moderately important  2PT+1FM = Least important |
| Families can be aware of and be involved in developing treatment plans. | 2PT+1FM+1CL= Most important  1FM+1CL= Moderately important  1 PT+1FM+1CL= Least important |
| **3b. Question 2: Factors influencing family involvement** | **Stakeholder Endorsement (n= 9)** |
| Involvement is influenced by the resources of families such as finances, accessibility of the clinic, time available based on job and other commitments, etc. | 1PT+1FM+1CL = Most important  2PT = Moderately important  2FM+2CL = least important |
| Involvement is influenced by each family’s culture around autonomy and support. | 2FM = Most important  1PT+2CL = Moderately important  2PT+1FM+1CL = Least important |
| Involvement is influenced by the age or development of patients (e.g., adolescents versus young adults versus adults). | 1FM+1CL = Most important  1PT+1FM+2CL = Moderately important  2PT+1FM = Least important |
| There should always be some involvement of families/carers in treatment. | 2FM = Most important  2PT+1FM = Moderately important  1PT+3CL= Least important |
| The frequency and types of involvement of families should be discussed jointly by patients, families and treating teams. | 1PT+1FM+2CL= Most important  2PT+1FM+1CL = Moderately important  1FM = Least important |
| **3c. Question 3: Contact frequency for family involvement** | **Stakeholder Endorsement (n= 9)** |
| Families should be present at every point in treatment. | 1PT+1FM+1CL = Most important  1FM+1CL = Moderately important  2PT+1FM+1CL = least important |
| Families and the treating team should have contact with each other at least once a week for the first month. | 2PT+2FM = Most important  1PT+1FM+2CL= Moderately important  1CL = Least important |
| Families and the treating team should have contact with each other at least once a month throughout treatment (which is usually for 2 years at *). | 1PT+2FM+1CL = Most important  2PT+1FM+1CL = Moderately important  1CL = Least important |
| Over the course of two years, **there should be** a minimum number of times that treatment teams should contact families. Beyond this minimum, treatment teams can also increase contact depending on patients’ needs. | 1PT+1FM+2CL= Most important  1PT+1FM+1CL+= Moderately important  1PT+1FM = Least important |
| There should be guidelines about involving families for treatment teams to follow. | 1FM+2CL = Most important  1PT+1FM+1CL = Moderately important  2PT+1FM = Least important |
| **3d. Question 4: Dealing with consent and confidentiality of family involvement** | **Stakeholder Endorsement (n= 9)** |
| If a patient is not a threat to himself or others, there should be no insistence on involving families/carers if the patient does not desire such involvement. | 2PT+1FM = Most important  1PT+1CL = Moderately important  2FM+2CL = least important |
| When the patient has consented for families to be involved, treatment providers should use their judgment in deciding what information should and should not be disclosed to families. | 1PT+1FM+2CL= Most important  1PT+1FM+1CL= Moderately important  1PT+1FM = Least important |
| It is possible to involve families/carers and also respect patient consent and confidentiality**.** | 2PT+1FM+1CL= Most important  1PT+1FM+2CL = Moderately important  1FM = Least important |
| Even when patients consent for families to be involved, patients themselves should make key treatment decisions. | 2CL = Most important  1PT+1FM+1CL= Moderately important  2PT+2FM = Least important |
| When patients consent for families to be involved, key treatment decisions should involve both the patient and the family. | 1PT +2FM = Most important  1PT+1FM+2CL= Moderately important  1PT+1CL = Least important |
| **Legend:** Ranks 1-3 = Most important, 4-7 = Moderately important, 8-10 = Least important  PT = Patient; FM = Family member; CL = Clinician ; *Name of the EIS redacted for confidentiality purposes | |
