# Supplementary material for: Exploring the preferences of multiple stakeholder groups for family involvement in early intervention services for psychosis: A cross-sectional mixed-methods study
Source: PLOS Ment Health. 2026 Apr 10;3(4):e0000430. doi: 10.1371/journal.pmen.0000430 (PMC13068328; doi:10.1371/journal.pmen.0000430)
Supplement: Attachment — Submitted filename: Response to reviewers_.docx [file pmen.0000430.s005.docx]

RESPONSE TO REVIEWERS

PMEN-D-25-00301
Manuscript: Exploring the preferences of multiple stakeholder groups for family involvement in early intervention services for psychosis using modified nominal group technique
Submitted to: PLOS Mental Health

Dear Editor,

We thank you and the reviewers for these comments which have strengthened our paper. All changes are highlighted in yellow in the revised paper for ease of reference. Below, we have responded to each comment in blue font.

1. Please provide additional details regarding participant consent. In the ethics statement in the Methods and online submission information, please ensure that you have specified (1) whether consent was informed and (2) what type you obtained (for instance, written or verbal, and if verbal, how it was documented and witnessed). If your study included minors, state whether you obtained consent from parents or guardians. If the need for consent was waived by the ethics committee, please include this information.

This information regarding consent is included in the paper.

2.If you are reporting a retrospective study of medical records or archived samples, please ensure that you have discussed whether all data were fully anonymized before you accessed them and/or whether the IRB or ethics committee waived the requirement for informed consent. If patients provided informed written consent to have data from their medical records used in research, please include this information.

This is not applicable.

Reviewer #1: The following are my suggestions for your manuscript.

1. Methodology is absent in the abstract.

We have now made the methodology in abstract clearer. It now reads: “*This study aimed to understand stakeholders’ views about involving families in care for youth experiencing early psychosis using a cross-sectional mixed method design. The study was conducted at an early intervention service for psychosis in Montreal. Nine participants (patients, family members, clinicians) were purposively recruited. They participated in a one-day workshop using nominal group technique, whose discussion was subjected first to thematic and then content analysis to develop statements/recommendations regarding family involvement, which were ranked by participants.*”

2. The study's title ought to be defined by its design; upon reading the content, the title appears mismatched. Please see Lines 1-3 for the modified title (*Exploring the preferences of multiple stakeholder groups for family involvement in early intervention services for psychosis using a cross-sectional mixed method design*)

3. At line 58, mention is made of there being no evidence; subsequently, please incorporate existing literature and identify the research gap.
4. Please incorporate existing literature.

Thank you for this comment. In the introduction section, we have now more thoroughly elaborated on the evidence base, adding relevant citations, for family involvement and interventions in first-episode psychosis; for implementation gaps in this regard; how families feel disregarded in psychosis care, and how Canadian clinical guidelines in early psychosis do not provide implementable recommendations for family involvement in care. We have also now more clearly drawn out the gap in this evidence base (around views of stakeholder groups, particularly integrating within one study multiple stakeholder groups; as well as generating concrete recommendations for family involvement from such stakeholder engagement), and our two objectives are aligned with these gaps.

5. Conceptual and operational clarity regarding family involvement is lacking within the introduction. We have now added a conceptualisation of family involvement in the introduction, alongwith the relevant citations, “*In mental health, family involvement can be seen as a continuum, encompassing an extension of the traditional function of the family (e.g., providing affection and encouragement, emotional and social support; housing, transportation, nutrition, and financial support), as well as additional caregiving-related responsibilities (facilitating help-seeking, attending appointments, encouraging treatment adherence, participating in treatment decisions and family interventions) and advocacy”.*

6. At line 66, alongside the research gap, please include the statement of the problem or the scope of the current study. As discussed above, we have now strengthened the introduction and presented more clearly the research gap. Furthermore, our statement about the problem or scope of the current study now reads (relevant citations are included in the manuscript), “*While there have been studies examining views on family involvement of individual stakeholder groups, there have been few if any attempts to integrate the views and preferences of multiple stakeholders (patients; families; and clinicians and clinician-decision-makers/program leaders) regarding the involvement of families in early intervention services. Gaps therefore remain in knowledge regarding how these stakeholders’ perspectives converge and diverge; how stakeholders currently navigate family work and associated tensions and decision-points (e.g., around confidentiality, balancing competing priorities for clinicians, etc.); and how they think these should be effectively navigated (i.e., their preferences)*.”

7. It would be beneficial were specific objectives or research questions added at the introduction's end. We have clarified the study objectives: *“Addressing these gaps, the aim of the study was to examine the perspectives of multiple stakeholder groups on family involvement in early intervention for psychosis. Specifically, the objectives of our study were (1) to explore in depth, the views and preferences of all pertinent stakeholder groups regarding the involvement of families in early intervention services (Phase 1); (2) to arrive at concrete recommendations for how often families should be involved over the course of their loved one’s follow-up, and how patient consent and confidentiality vis-à-vis the involvement of their families should be navigated*.”

8. I believe readers will find the methodology section confusing, as it appears disorganized; subheadings such as study design, sampling method and estimation, tools for data collection, data collection process, data analysis, and ethical statement would be appreciated. These headings and subheadings in the Methods section have been presented and titled as per the journal requirement (font size, bold, and numbered). The ethics statement is presented as a separate section at the end of the study design section, entitled “Statement about ethics”: “*Ethics approval was obtained from the research ethics board of the Douglas Mental Health University Institute (IUSMD-19-34). All participants were adults and provided written informed consent for participation and audio-recording of the discussion.*”

9. At line 627, please include reliability and validity information. The e-survey is not a measure with psychometric properties in the traditional sense. Instead, aligned with nominal group technique, it is a series of statements derived from the discussion among stakeholders, which were then ranked by participants. Thus, reliability and validity are not pertinent in this case.

10. Overall, there ought to be proper specific objectives; results should align with these, and discussion should follow accordingly, lest readers become confused. The document is excessively lengthy; hence, my suggestion is that sequencing would improve it. We have now reduced the word length of the manuscript and made it concise. The introduction now ends with an overall aim and 2 specific objectives. Our data analysis and results section are clearly organized around these two objectives. We hope this has strengthened the clarity of the manuscript.

11. The e-survey is somewhat perplexing; please align it with specific objectives. The e-survey is related to Objective 2 and phase 2 of the study. This has now been clarified in each section of the study for ease of reading.

12. Integrated findings and a triangulation method are absent from the manuscript. We have now more clearly presented our study design and clarified how findings are first presented separately and then triangulated in the discussion stage. We have also described the triangulation method under 2.5 Data analysis. “*Data collected from the two phases were analysed sequentially and are presented separately in the Results, with findings from both phases triangulated in the Discussion to arrive at a cohesive picture. Triangulation involved comparing and cross-checking results from the two phases to identify patterns and areas of agreement, while also drawing on Phase 1 for nuanced insights and Phase 2 for concrete recommendations*”. Later in the same section we added: “*Results generated from the qualitative and quantitative methods used were integrated to form an overall interpretation of stakeholder views on the role of families in the lives and treatment of young persons with psychosis, and their recommendations for involving families in early intervention services for psychosis, which is presented in the Discussion*.”

13. Limitations are poorly presented; they should be articulated in a specific and critical manner. The limitations have now been presented more critically, and organized thematically, under those stemming from sampling and setting, and those stemming from methods and design.

14. Recommendations for further research are lacking. Thank you for this suggestion. We have now added some recommendations: “*Future work should replicate this study across diverse early intervention services, which may surface additional facets of family involvement and strengthen generalisability. These recommendations could be considered for integration in clinical practice guidelines, as a top-down directive for family involvement in psychosis care may carry more “weight” than families constantly having to advocate for themselves.”*

Reviewer #2: The manuscript makes a timely and valuable contribution to understanding stakeholder perspectives on family involvement in early intervention for psychosis. Strengths include:

Use of a participatory, consensus-driven methodology.

Inclusion of multiple stakeholder groups (patients, families, clinicians).

Integration of qualitative themes with ranking-based prioritization to generate actionable recommendations.

Careful consideration of sensitive issues such as consent and confidentiality.

Thank you for your encouraging and positive feedback.

Areas for improvement:

1.Sample size and representativeness – With only nine participants from a single Canadian service, findings should be presented as exploratory. Please discuss limitations in terms of generalizability.-- We have reorganised our limitations and mention: “*Nonetheless, our study has important limitations stemming from our sampling and setting, and from our methodology/design. Our small sample, while appropriate for the method, limits diversity in family structures and cultural contexts, and findings should therefore be treated as exploratory. Furthermore, this study included stakeholders from a single early intervention service in a Canadian city which limits the transferability of our findings to other contexts where different organizational, cultural, and societal factors may influence experiences of young people, families, and clinicians. Further, our study only included consenting patients and families who were or had been engaged in care in the early intervention services. It does not include the perspectives of those who disengaged from treatment and/or those who did not want their families to be involved at all in their care while they were in treatment.* ”

2.Data availability – Clarify what type of de-identified data (e.g., excerpts, coding framework, survey statements) could be shared in line with ethical restrictions. We have clarified that, “Survey statements and their ranking are available as supplementary material. Deidentified transcripts cannot be shared as participants did not give consent for sharing these, but may be available (e.g., as themes, analyzed locally and then findings shared) to inform secondary qualitative data analysis projects by contacting the author*.* ”

3.Recommendations table – Table 4 lists consensus recommendations, but more detailed discussion on how these could inform clinical guidelines would strengthen impact. Our discussion has been modified to reflect this suggestion, and now starts with this: “*Family-focused recommendations included in guidelines/standards for first episode psychosis have mostly focused on aspects of direct care, pertaining to involving families in assessment and treatment planning. However, these recommendations have been criticized for their lack of pragmatic and concrete guidance and for not addressing critical issues. Some examples of unaddressed issues include navigating barriers to involvement, handling consent and confidentiality, and specifying concrete modes of family involvement (e.g., frequency, modality). Moreover, previous guidelines have shown low real-world applicability, likely due to their disconnection from the values, preferences and perspectives of families, patients and clinicians. To address these gaps, we listed several recommendations based on participants' contributions and consensus, covering specific circumstances under which family involvement is considered crucial (crises, relapses, hospitalizations), ways how families may provide support (e.g.: treatment adherence and adjustment), and guidance to navigate and negotiate consent issues and systematically record contacts between families and treatment teams*.”

4.Language/clarity – Some sections could be streamlined (e.g., Results subsections are very long with extensive quotes). Consider condensing while retaining key illustrative examples. Thank you for this feedback. We have made many edits throughout the manuscript, including Results section (shortening quotes, choosing quotes) for ease of reading.

5.Future directions – The discussion could highlight next steps, such as replicating the study with larger and more diverse samples, or integrating findings into policy frameworks. Please see the last paragraph in conclusion section “*Future work should replicate this study across diverse early intervention services, which may surface additional facets of family involvement and strengthen generalisability. These recommendations could be considered for integration in clinical practice guidelines, as a top-down directive for family involvement in psychosis care may carry more “weight” than families constantly having to advocate for themselves.* ”

6.Overall, I believe this is a strong and worthwhile paper suitable for publication after minor revisions.

Thank you for this positive appraisal.
